# Superconductivity assisted change of the perpendicular magnetic anisotropy in V/MgO/Fe junctions

**DOI:** 10.1038/s41598-021-98079-5

**Published:** 2021-09-24

**Authors:** César González-Ruano, Diego Caso, Lina G. Johnsen, Coriolan Tiusan, Michel Hehn, Niladri Banerjee, Jacob Linder, Farkhad G. Aliev

**Affiliations:** 1 Departamento Física de la Materia Condensada C-III, Instituto Nicolás Cabrera (INC) and Condensed Matter Physics Institute (IFIMAC), Universidad Autónoma de Madrid, Madrid, 28049 Spain; 2 Department of Physics, Center for Quantum Spintronics, Norwegian University of Science and Technology, 7491 Trondheim, Norway; 3 Department of Physics and Chemistry, Center of Superconductivity Spintronics and Surface Science C4S, Technical University of Cluj-Napoca, Cluj-Napoca, 400114 Romania; 4 Institut Jean Lamour, Nancy Universitè, 54506 Vandoeuvre-les-Nancy Cedex, France; 5 Department of Physics, Loughborough University, Epinal Way, Loughborough, LE11 3TU UK

**Keywords:** Condensed-matter physics, Ferromagnetism, Magnetic properties and materials, Spintronics, Superconducting properties and materials, Materials science, Materials for devices, Information storage, Nanoscience and technology, Magnetic devices, Superconducting devices

## Abstract

Controlling the perpendicular magnetic anisotropy (PMA) in thin films has received considerable attention in recent years due to its technological importance. PMA based devices usually involve heavy-metal (oxide)/ferromagnetic-metal bilayers, where, thanks to interfacial spin-orbit coupling (SOC), the in-plane (IP) stability of the magnetisation is broken. Here we show that in V/MgO/Fe(001) epitaxial junctions with competing in-plane and out-of-plane (OOP) magnetic anisotropies, the SOC mediated interaction between a ferromagnet (FM) and a superconductor (SC) enhances the effective PMA below the superconducting transition. This produces a partial magnetisation reorientation without any applied field for all but the largest junctions, where the IP anisotropy is more robust; for the smallest junctions there is a reduction of the field required to induce a complete OOP transition ($H_\text {OOP}$) due to the stronger competition between the IP and OOP anisotropies. Our results suggest that the degree of effective PMA could be controlled by the junction lateral size in the presence of superconductivity and an applied electric field. We also discuss how the $H_\text {OOP}$ field could be affected by the interaction between magnetic stray fields and superconducting vortices. Our experimental findings, supported by numerical modelling of the ferromagnet-superconductor interaction, open pathways to active control of magnetic anisotropy in the emerging dissipation-free superconducting spin electronics.

## Introduction

Control of out-of-plane (OOP) anisotropies in ultra thin ferromagnetic multilayer films have revolutionized magnetic storage and spintronics technologies by mitigating the impact of the demagnetizing energy as the bit and magnetic tunnel junction sizes diminished^1,2^. Tuning of perpendicular magnetic anisotropy (PMA) by careful selection of structure design^3,4^ and size^5^ has been among the main challenges of spintronics. Besides the variation of the ferromagnet thickness and interface with oxides, OOP magnetisation reorientation can be achieved by a temporary reduction of the IP-OOP barrier using, for example, heat and microwave pulses^6,7^ or a combination of magnetic field and low temperature^8^.

Recently, we demonstrated a fundamentally different route to magnetisation reorientation through the influence of superconductivity on the IP magnetisation anisotropy^9^. The key idea behind this effect is that the magnetisation aligns to minimize the weakening of the superconducting condensate associated with the creation of spin triplet (ST) Cooper pairs^10^. The spin triplet generation depends on the magnetisation direction relative to the interfacial Rashba spin-orbit field. Understanding the factors influencing this superconductivity-induced change of magnetic anisotropy is crucial for designing the next generation of cryogenic memories in the emerging field of superconducting spintronics, where control over non-volatile magnetisation states still remains a major challenge^11–14^.

The main underlying physical mechanisms for the transformation of ST Cooper pairs from singlet to mixed-spin and equal-spin triplet pairs are magnetic inhomogeneities^15,16^, two misaligned FM layers^17,18^ or SOC^19^. Previous experiments focusing on SOC-driven generation of triplets have focused on heavy metal (Pt) layers in non-epitaxial SC/FM structures^20,21^ and Rashba SOC in epitaxial V/MgO/Fe junctions^9,22^ where ST Cooper pairs are generated depending on the magnetisation orientation relative to the Rashba field.

Theoretically, it has been shown^10^ that a superconductor coupled to a ferromagnet by SOC could stimulate the modification not only of the IP^9^, but also of the OOP magnetic anisotropy below the superconducting critical temperature ($T_C$). Due to the stray fields, however, ferromagnetic films are expected to have a stronger interaction with the superconductor when an OOP magnetisation is present, compared to a simple IP variation^23,24^. Therefore, a careful consideration of the interaction of these stray field generated by the OOP magnetisation and superconducting vortices is essential to fully capture the factors influencing the effective OOP anisotropy.

Here, we investigate the superconductivity-induced OOP magnetisation reorientation in epitaxial Fe(001) films with competing IP and OOP anisotropies, both at zero field and in the presence of out-of-plane applied magnetic fields. The V/MgO/Fe(001) junctions are ideal candidates to verify the predicted modification of the effective perpendicular anisotropy in the superconducting state for several reasons^10^. Firstly, the Fe(001) has the required^10^ cubic symmetry; secondly, previous studies show that the normal state IP-OOP reorientation transition takes place at a well-defined critical field^8^; thirdly, the system has Rashba type SOC, which is responsible for the PMA in MgO/Fe^25^; fourthly, the relative contribution of the IP and OOP magnetisation anisotropies can be tuned by changing the junction lateral size, and SOC can be varied by applying an external electric field; finally, the change in magnetisation can be determined with high precision by studying the transport characteristics using a second magnetically hard Fe/Co layer which is magnetostatically decoupled from the soft Fe layer^8^.

For the smallest junctions, where the IP and OOP anisotropies strongly compete, we remarkably observe the full superconductivity-induced IP-OOP magnetisation reorientation predicted in Ref.^10^. This results in (i) a decreasing of the required field to induce the full IP-OOP transition below $T_C$, which is not observed in bigger junctions; and (ii) a spontaneous increasing of the misalignment angle between the two FM layers below $T_C$ in the absence of applied field, which is consistently observed in all but the largest junctions. These differences in the observed behaviour depending on the junctions dimensions are most likely due to the IP anisotropy becoming more dominant with increasing lateral size. We discard the magnetostatic interaction between supeconducting vortices and the FM layers as the main cause of the observed effects.

## Results

**Figure 1:**
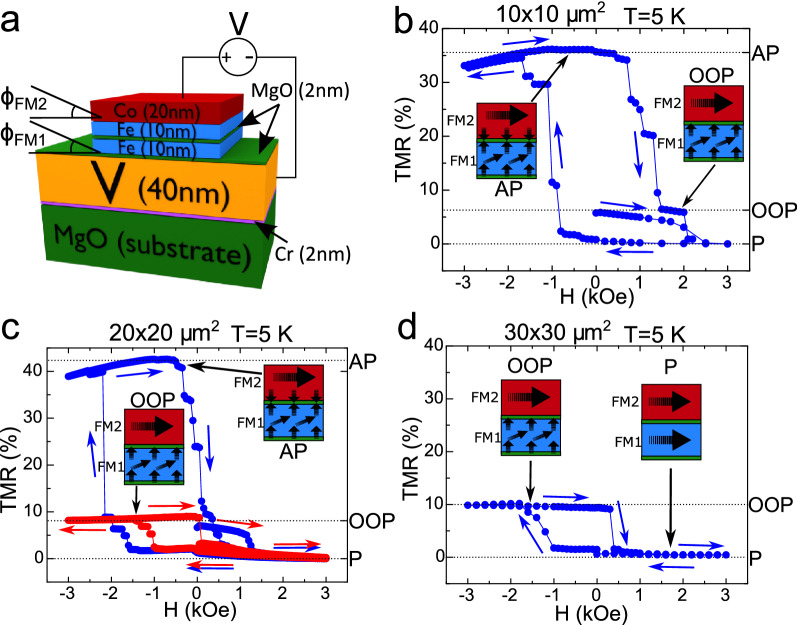
(**a**) Sketch of the junctions under study where Fe(10 nm) (FM1) is the soft ferromagnet undergoing spin reorientation transitions, while Fe(10 nm)Co(20 nm) (FM2) is the hard (sensing) layer. $\phi _\text {FM1}$ and $\phi _\text {FM2}$ are the OOP angles of each FM layer (i.e. the angle with respect to the plane of the layers). Since the FM2 layer is normally fixed to act as a sensor, $\phi _\text {FM2}$ is assumed to be very close to 0 unless otherwise stated. (**b**–**d**) show TMR experiments where the field is applied in the OOP direction in $10\times 10$, $20\times 20$ and $30\times 30$ $\upmu \text {m}^2$ junctions respectively, showing the field-induced transition into the nonvolatile OOP state. The right vertical axes indicate the parallel (P), antiparallel (AP) and OOP states for each junction, marked with dotted lines. The inset sketches depict the proposed configuration of the two FM layers in the P (only shown in panel (**d**)), OOP and AP configurations of the spin valve stack.

Figure 1 shows the experimental configuration and the different types of OOP transition observed above the vanadium $T_C$. Figure 1a shows the V(40 nm)/MgO(2 nm)/Fe(10 nm)/MgO(2 nm)/Fe(10 nm)/Co(20 nm) (N(SC)/FM1/FM2) junctions with a hard Fe/Co layer (FM2) sensing the magnetisation alignment of the 10 nm thick Fe(001) soft layer (FM1). Details about the sample growth, characterization and the experimental set-up are explained in the “Methods” section. All junctions were saturated with a 3 kOe IP magnetic field (see the alignment calibration procedure in Supplementary Material, Sect. 1) before each of the OOP magnetoresistance (TMR) measurements, in order to eliminate magnetic inhomogeneities from previous OOP measurements. All except one of the studied junctions showed OOP anisotropy below 3 kOe. On the right side of the vertical axes of Fig. 1b–d, we indicate the TMR values corresponding to the well established parallel (P), perpendicular out-of-plane (OOP) and antiparallel (AP) states for each sample, which are used to calibrate the angle between the two FM layers ($\Delta \phi =\phi _\text {FM1}-\phi _\text {FM2}$, where $\phi _\text {FM1}$ and $\phi _\text {FM2}$ are the angles of each FM layer with respect to the plane of the layers, as shown in Fig. 1a) with the same procedure as described in Refs.^8,9^. This indicates that the IP-OOP transition also triggers a total or partial reorientation of the sensing (hard) FM2 layer, providing a resistance close to that of an AP state. Previous OOP measurements^8^ above $T_C$ made on only two $20\times 20$ $\upmu \text {m}^2$ junctions revealed asymmetric transitions into the perpendicular alignment of the soft FM1 layer, without any subsequent transition of the sensing layer with perpendicular fields up to 3 kOe. The present study is made with a total of 16 junctions of four different lateral sizes, where about half of them also demonstrate a transition to an AP configuration when the magnetic field is further increased after the transition to the OOP state has been completed. This AP configuration could potentially be either with the two layers oriented OOP or IP, although it seems rather unlikely that both layers reorient to an IP configuration while the applied OOP field increases. We believe that the high-field-induced transition from OOP to AP alignment or, in some cases, a nearly direct P to AP transition in N/FM1/FM2 junctions could be a consequence of the enhanced antiferromagnetic coupling reported for MgO magnetic tunnel junctions with perpendicular magnetic anisotropy (see^26^). We cannot exclude the possibility that the AP alignment could be triggered by a partial reorientation of the hard Fe/Co layer (with only the Fe part or the atomic layers closer to the Fe/MgO interface in the hard layer orienting OOP, as shown in the sketches in Fig. 1b,c). However, since we measure the total resistance of the junctions, it is impossible to distinguish between these two cases from transport measurements alone. Therefore, we mainly focus on the influence of superconductivity on the transition between IP and OOP states and the partial OOP reorientation at zero magnetic field. Consequently, for the OOP field range reported here, we will assume that the $\phi _\text {FM2}$ angle of the FM2 layer with respect to the in-plane configuration is fixed and close to 0.

Figure 1c shows typical OOP TMR cycles measured in two $20\times 20$ $\upmu \text {m}^2$ junctions, one of them switching to an AP alignment following an OOP orientation (blue) and the other one only switches to the OOP state (red). Figure 1d shows an OOP TMR for a $30\times 30$ $\upmu \text {m}^2$ junction where the OOP alignment of the FM1 and FM2 electrodes remains stable up to 3 kOe. Note that all junctions showed remanent OOP alignment of the soft Fe(001) electrode once the perpendicular magnetic field is removed (Fig. 1). This indicates the relatively small number of interfacial defects present in our junctions, as supported by numerical simulations analyzing the OOP configuration robustness as a function of the density of interfacial defects by studying the *inverse* OOP to IP transition, which are discussed in the Supplementary Material, S2.2.

The symmetry broken spin reorientation observed in the OOP TMR experiments shown in Fig. 1 b–d, has been previously explained in Ref.^8^ by the difference in the dislocation density present at the top and bottom surfaces of the soft Fe(001) layer due to the growth process. This differently affects the top and bottom surface anisotropies, which leads to different intensities at each interface resulting in the magnetisation being more easily reoriented into the OOP configuration for one field direction than the other. This asymmetric field behaviour might seem at odds with the Stoner–Rashba model developed in Ref.^27^. This model suggests that a net Rashba field related to the asymmetric top and bottom interfaces of a ferromagnetic film leads to a pseudo-dipolar contribution to the anisotropy, which would mainly favor an in-plane magnetisation and an uniaxial-like anisotropy favouring the/a perpendicular magnetisation configuration. Correspondingly, the hysteresis curve of a single magnetic (here Fe) layer is expected to be an even function with respect to the external magnetic field. However, we note that the model does not fully account for the complexities discussed below that could lead to the asymmetric hysteresis we observe in our multilayer structures.

The fact that the hysteresis curve is not an even function of the external magnetic field is simply related to the fact that the model is developed for a single ferromagnetic layer while in our complex heterostructure, we do not reverse the Fe/Co interface magnetisation. This is not unreasonable considering a large interface anisotropy. A full magnetisation reversal including interfacial magnetisation would only result in an asymmetric hysteretic response. Secondly, in our structures stray fields play a relevant/crucial/central role and importantly the stray fields seen by both interfaces are not similar. The bottom interface experiences the stray field of the Fe/Co top bilayer, while the top interface sees the contribution from the bottom Fe layer. In a macrospin model, increasing the stray fields would decrease the perpendicular anisotropy. To fully understand the complexities of the asymmetric magnetisation response, future studies such as direct OOP magnetisation measurements on the MgO/Fe/MgO structures in the absence of the sensing Fe/Co and V/MgO layers, could be performed.

It is worth mentioning a distinct feature of our junctions, having a strongly preferred IP magnetisation at room temperature^8^, with the OOP configuration of the soft 10 nm Fe layer only becoming non-volatile below 80 K. In the temperature range in which this study takes place (0.3 to 7 K), the magnetic field required to induce an OOP transition in the soft layer does not typically exceed 2 kOe. These relatively low values (with respect to continuous 10 nm thick Fe films) could be explained by the combined influence of a few factors. Firstly, the variation of the relation between the IP and OOP anisotropy energies could vary with temperature, possibly favouring the OOP configuration at low temperatures^28^. Secondly, interfacial strain has also demonstrated the potential to induce changes in the perpendicular anisotropy in thin ferromagnetic films^29^. Thirdly, the IP saturation in this study was carried out with a field of 3 kOe. This value was considered sufficiently high since the resistance values were stable above 1 kOe, but it could be insufficient to induce a perfect IP alignment at low temperatures. This factor could be more relevant for the smallest junctions where edge magnetic charges would have a relatively higher influence on the measured OOP switching field, qualitatively explaining the dependence of this field with the junctions lateral size, as supported by numerical simulations (see Supplementary Material Fig. S5). Finally, as mentioned before, as long as we measure the total resistance of the junction, we can’t exclude that the OOP reorientation might take place preferently in the atomic layers closer to the Fe/MgO interface (where it would be easier to reorient the magnetic moments due to the surface anisotropy). Thus, the surface OOP state (with a thickness of a few nm, close to that of the Fe magnetic exchange length^30^) might be realized with the aid of interface anisotropy at the Fe/MgO interface and an external OOP magnetic field. This is shown in the sketches of the spin valve configuration in Fig. 1b–d.

### Superconductivity induced change of the out-of-plane anisotropy field

**Figure 2:**
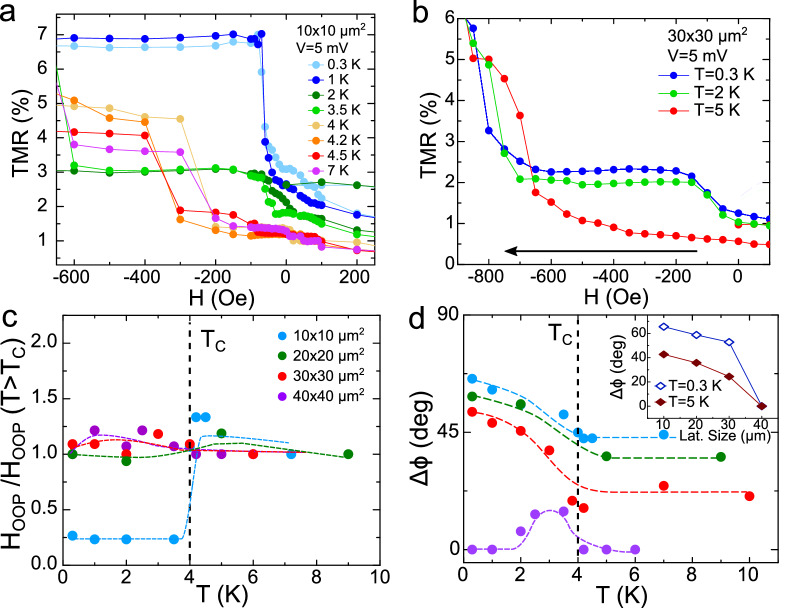
(**a**) Field induced OOP magnetisation transition in a $10\times 10$ $\upmu \text {m}^2$ SC/FM1/FM2 junction at different temperatures from above to below $T_C$. A strong reduction of $H_\text {OOP}$ takes place below $T_C$. (**b**) Shows a similar experiment in a $30\times 30$ $\upmu \text {m}^2$ junction. In this case, some increasesting in the low field TMR is observed, but not enough to be attributed to a complete OOP reorientation. (**c**) Temperature dependence of the normalized $H_{\text {OOP}}$ anisotropy field for junctions with four different lateral sizes. (**d**) Represents the temperature dependence of the misalignment angle between the two FM layers ($\Delta \phi =\phi _\text {FM1}-\phi _\text {FM2}$, calibrated following the procedure outlined in Ref.^9^) at zero field for the four different sized samples, using the same color legend as in (**c**). The inset shows a comparison of the zero field $\Delta \phi$ angle at $T=5$ K (above $T_C$) and at $T=0.3$ K (well below $T_C$) as a function of the samples’ lateral size. The gradual decrease of the zero-field angle above $T_C$ with increasing lateral size points towards a small equilibrium initial angle already existing in the normal state, which we attribute to competing OOP and IP anisotropies. When superconductivity develops below $T_C$, an additional magnetisation reorientation is observed in all except the bigger samples. The colored dashed lines are guides for the eyes, while the vertical, black, dashed lines indicate the critical temperature.

Figure 2a shows the temperature dependence of OOP TMRs in a $10\times 10$ $\upmu \text {m}^2$ junction, in a field range where the field-induced magnetisation reorientation of the FM1 layer (measured at 5 mV) takes place. A decrease of the characteristic $H_{\text {OOP}}$ field (defined as the applied magnetic field providing a *complete* OOP reorientation of the FM1 layer) just below $T_C$ can be observed upon lowering the temperature, as represented in Fig. 2c. We note that the SC-induced full IP-OOP transitions have been clearly observed in the smallest $10\times 10$ $\upmu \text {m}^2$ lateral size junctions. The larger junctions showed a small low field TMR increase below $T_C$, which could be interpreted either as a partial FM1 layer reorientation or an inhomogeneous OOP alignment (Fig. 2b). For the $20\times 20$ $\upmu \text {m}^2$ and larger junctions, the $H_{\text {OOP}}$ anisotropy field turned out to be nearly independent of temperature (Fig. 2c). Interestingly, our junctions also revealed spontaneous zero field TMR emerging below $T_C$ (corresponding to a *partial* magnetic reorientation of the soft FM1 layer), which is more pronounced for the smaller samples and diminishes with lateral size, abruptly disappearing for the largest junctions. This is shown in Fig. 2d, where instead of the TMR, the calculated angle between the two FM layer is plotted. It is worth noting that this relative angle calculation is similar to our previous work^8,9^, and assumes a uniform magnetisation in the whole FM layer. However, the real scenario could be more complex (see Supplementary Material, S2.1).

### Influence of electric field on the out-of-plane reorientation

The presence of the MgO barriers allows us to explore the possible influence of high electric fields on the magnetic-field-induced IP-OOP transitions above and below $T_C$. High electric field influences the PMA anisotropy by modifying the SOC Rashba field in magnetic tunnel junctions^27^. Our previous study^8^ revealed that roughly two thirds of the voltage drop in our junctions occurs at the V/MgO/Fe barrier, resulting in a high electric field across this interface. The remaining voltage drops at the Fe/MgO/Fe interface, which is responsible for the change in the resistance providing the measured TMR depending on the relative magnetic configuration of the two FM layers.

**Figure 3:**
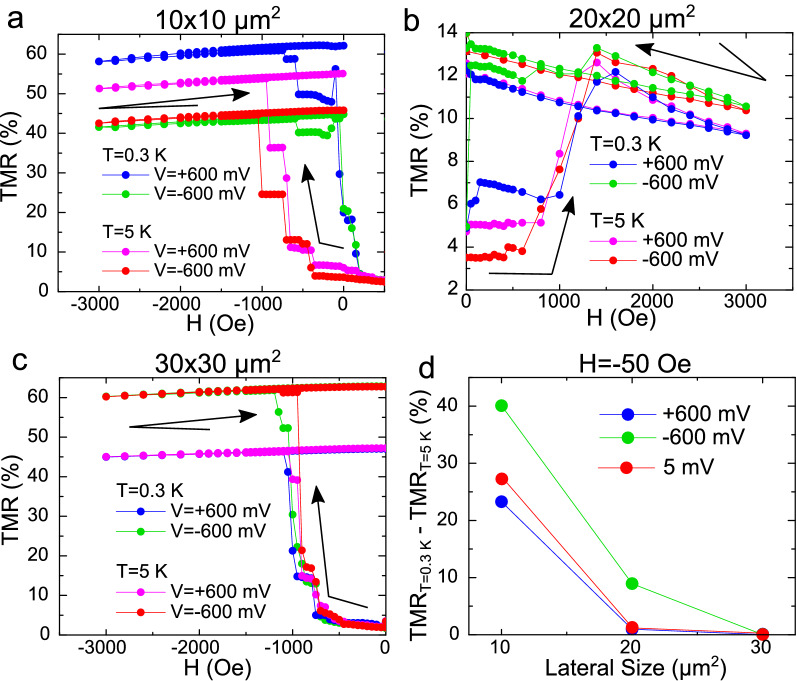
Influence of the electric field on the magnetisation reorientation transition $H_{\text {OOP}}$, above ($T=5$ K) and below $T_C$ ($T=0.3$ K). The transition is shown for an applied bias of 600 mV (electric field of about $2.5\times 10^8$ V/m), with both positive and negative polarities, for samples with varying lateral sizes: (**a**) $10\times 10$ $\upmu \text {m}^2$, (**b**) $20\times 20$ $\upmu \text {m}^2$ and (**c**) $30\times 30$ $\upmu \text {m}^2$. (**d**) shows the difference of TMR with temperature (calculated as $\text {TMR}_{0.3\text { K}}-\text {TMR}_{5\text { K}}$) for both polarities and in the absence of applied electric field ($V=5$ mV) as a function of the lateral size, for an applied field of $H=-\,50$ Oe. The superconducting transition seems to have bigger effects on the magnetic OOP reorientation for smaller samples.

We have therefore investigated the influence of high bias and its polarity on the IP-OOP transition in junctions with different lateral sizes. Figure 3a–d show that an applied bias of 600 mV (generating an electric field at the V/MgO/Fe interface exceeding $2\times 10^8$ V/m) hardly affects $H_{\text {OOP}}$ above $T_C$, independently of the junctions size. Moreover, the application of a large electric field has also a negligible effect on the superconductivity-induced IP-OOP transition in the larger than $30\times 30$ $\upmu \text {m}^2$ junctions, with a dominant IP magnetisation alignment (Fig. 3c). However this changes for the smaller junctions, where IP and OOP anisotropy values are comparable leading to an entirely different behaviour. Strikingly, we observe that for $10\times 10$ $\upmu \text {m}^2$ and $20\times 20$ $\upmu \text {m}^2$ junctions, the electric field stimulates an IP-OOP transition below $T_C$ at very small values of the applied magnetic field (below 100 Oe).

Figure 3d compares the influence of an electric bias close to 600 mV with different polarities on the magnetisation alignment below $T_C$ (0.3 K) with an applied magnetic field of $-\,50$ Oe, within the field range in which we observed a larger influence of the electric field on the IP-OOP transition for the smaller junctions. This field is about an order of magnitude below the first critical field of our Vanadium films, which was estimated to be close to 400 Oe^22^, therefore minimizing the presence of vortices in the superconducting layer. We believe that the electric field effect asymmetry could be due to the combined influence of the relatively more dominant proximity effects between the SC and FM states at the V/MgO/Fe interface in smaller junctions, and the electric-field-induced variation of the Rashba field influencing the OOP anisotropy for the non-equivalent interfaces MgO/Fe and Fe/MgO in the junctions^27^.

## Discussion

### Evaluation of magnetostatic coupling between superconducting vortices and ferromagnet

Let us start our discussion by considering different scenarios involving the possible magnetostatic coupling between the superconducting vortices and the ferromagnet^31^. It is tempting to consider the device edges as mainly responsible for the superconductivity-induced spin reorientation, as the edge has a more important contribution for the smallest samples, in which the minimum applied field is enough to fully reorient the magnetisation. However, a few experimental facts contradict this scenario. Firstly, the superconductivity-induced additional zero field OOP angle variation is similar for $10\times 10$ to $30\times 30$ $\upmu \text {m}^2$ junctions (see inset in Fig. 2 d), which would not be the case if the change comes from the device’s edges. The superconductivity induced spin reorientation effect abruptly diminishes for the $40\times 40$ $\upmu \text {m}^2$ junction only (Fig. 2d). Secondly, numerical simulations show that the OOP reorientation due to magnetostatic coupling, if relevant, could potentially be triggered by the nucleation of OOP domains in the interior of the samples rather than at the edges; even if we assume the edges as the initial OOP nucleation places, the resulting vortex distribution would affect the whole FM layer (see Supplementary Material S2.1). Finally, electric field stimulates the OOP transition for relatively small junctions with competing anisotropies (see Fig. 3) which points towards the possible role of the Rashba field.

We have seen from micromagnetic simulations and as an experimental trend that, on average, the normal state $H_{\text {OOP}}$ increases with the junctions area (Supplementary Material S2.3). This is in agreement with the gradual decrease of the partial OOP magnetisation reorientation with increasing lateral size seen in the normal state, just above the critical temperature (see Fig. 2d). Within the above picture, a lower $H_{\text {OOP}}$ field is required to reorient the magnetisation perpendicularly in the smallest junctions, and therefore one would expect a weaker magnetostatic coupling to SC vortices.

Numerical simulations of the magnetostatic interaction of the V/MgO/Fe system during an OOP TMR experiment such as the ones shown in Fig. 1, where a varying OOP magnetic field is applied, is a complex problem which requires self-consistent treatment of the interaction between magnetic charges and stray fields of superconducting vortices^32^. The Supplementary Material (Sect. 2.1) introduces a simplified simulation scheme which evaluates this interaction in the presence of the Meissner effect. These results show that the vortex-mediated magnetostatic interaction might only explain a weak enhancement of $H_{\text {OOP}}$ in the superconducting state in the largest junctions (Fig. 2c). However, we note that varying the superconducting hysteresis strength or width in the magnetostatic simulations could not explain the strong decrease of $H_{\text {OOP}}$ below $T_C$ which was experimentally observed in the smaller junctions. Moreover, a dominant magnetostatic coupling would contradict the observed influence of electric field on TMR below $T_C$ for the smallest junctions (Figs. 2, 3).

### Microscopic model

To explain the strong decrease in the OOP anisotropy field below $T_{C}$ for the smallest junctions and the superconductivity-induced zero field magnetic reorientation in all except the largest ones, as well as the influence of the SOC strength through the application of an electric field, we present a microscopic model describing the observed superconductivity-assisted OOP magnetic reorientation. In heterostructures consisting of superconducting and magnetic layers, the superconducting condensate is weakened as Cooper pairs leak into the magnetic regions^33^. This leakage is more efficient when the spin-singlets are transformed into equal-spin triplet pairs polarized along the same axis as the magnetisation. In our system, the MgO layer boosts the Rashba SOC at the SC/FM interface allowing for a generation of equal-spin triplets that depends on the orientation of the magnetisation with respect to the interface^10,20^.

To show how the efficiency of the triplet leakage affects the critical field for reorienting the magnetisation OOP, we calculate the free energy of the system from a tight-binding Bogoliubov–de Gennes (BdG) Hamiltonian (see Sect. 3 in the Supplementary Material for a complete description of our method). The V/MgO/Fe structure is modelled as a cubic lattice with electron hopping between neighboring sites. We include conventional *s*-wave on-site superconducting pairing potential in the V layer, Rashba SOC in the MgO layer, and an exchange splitting between spins in the Fe layer. Although this model is valid in the ballistic limit, we expect similar results for diffusive materials since spin singlets are partially converted into odd-frequency *s*-wave triplets that are robust to impurity scattering. Moreover, the variation in the singlet population under IP to OOP reorientation of the magnetisation have previously been demonstrated both experimentally and by dirty limit calculations^20^.

The free energy determined from this model captures the contribution from the superconducting proximity effect, and also includes a normal-state contribution favoring an IP magnetisation. In addition, we include a normal-state anisotropy $K_{\text {IP}}[1-\cos ^4(\Phi _{\text {FM}1})]+K_{\text {OOP}}[1-\sin ^2 (\Phi _{\text {FM}1})]$, where $\Phi _{\text {FM}1}$ ranges from $0^{\circ }$ (corresponding to an IP magnetisation of the soft ferromagnet) to $90^{\circ }$ (OOP magnetisation). In total this gives a normal-state anisotropy favoring an IP magnetisation, with an additional local minimum for the OOP magnetisation direction. Here we only focus on the superconductivity-assisted deepening of these OOP quasi-minima associated with the spin singlet to spin triplet conversion. The increase in $H_{\text {OOP}}$ below $T_{C}$ discussed in the previous section is not covered by this theoretical framework, as it does not take into account formation of vortices or the size of the junction. The discussion here is therefore relevant to the smaller junctions where the superconductivity-assisted decrease in $H_{\text {OOP}}$ dominates.

**Figure 4:**
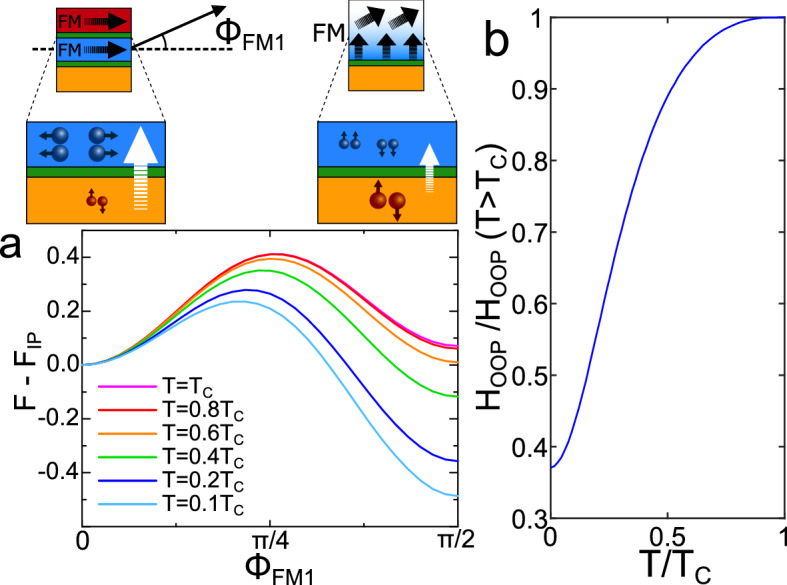
When the magnetisation of the soft ferromagnet is rotated from a parallel to an OOP alignment with respect to the hard ferromagnet, as sketched above (**a**) (In the upper right part, only the part of the soft FM layer closer to the V is depicted, not to scale, in order to show the possible magnetisation configuration. In the theoretical modelling the magnetisation is considered to be uniform for simplicity, altough, as mentioned before, experimentally the magnetisation reorientation is more likely to happen only close to the interface. The FM2 layer is considered to be fixed with an IP orientation), the SOC assisted conversion (white arrows) of singlet Cooper pairs (orange) into equal-spin triplets (blue) is at its minimum for the OOP orientation. The superconducting condensate is therefore stronger when the magnetisation is OOP, causing a decrease in the OOP free energy as the temperature is decreased below $T_C$ (panel a)). The deepening of the OOP minimum causes a decrease in $H_{\text {OOP}}$ (**b**)). In (**a**), $K_{\text {IP}}=1.5$ and $K_{\text {OOP}}=1.6$, while in (**b**) $K_{\text {anis}}=0.8$ favoring the IP orientation. The free energy is scaled by the hopping parameter *t*. For further details about the parameters used in the BdG calculations, see Sect. 3 in the Supplementary Material.

In Fig. 4a), we demonstrate how the local free energy minimum for an OOP magnetisation deepens as the temperature is decreased below $T_C$. As a simple qualitative model, we calculate the external magnetic field that can be used to force the magnetisation into the OOP orientation as $H_{\text {OOP}}=(K_{\text {anis}}+F_{\text {OOP}}-F_{\text {IP}})/\mu _0 \mu _{\text {tot}}$, where $\mu _{\text {tot}}$ is the total magnetic moment, $K_{\text {anis}}$ is a constant anisotropy favoring the IP orientation that includes the above mentioned parameters for the normal-state IP and OOP anisotropies, $K_\text {IP}$ and $K_\text {OOP}$, as well as an energy barrier associated with the reorientation; and $F_{\text {OOP}}$ and $F_{\text {IP}}$ are the calculated free energies in the OOP and IP states of the soft layer respectively. In Fig. 4b, we show how $H_{\text {OOP}}$ decreases below $T_C$ as observed for the $10\times 10$  $\upmu\hbox {m}^2$ junction in Fig. 2c. We have thus demonstrated that the proximity effect enables a strong decrease in $H_{\text {OOP}}$ that cannot be explained by the coupling of the ferromagnet to superconducting vortices discussed in the previous section. Moreover, since this variation in $H_{\text {OOP}}$ requires that SOC is present, it also explains the dependence on the electric field observed for the smaller junctions (Fig. 3). The fact that $H_{\text {OOP}}$ decreases over a longer temperature interval than in the experiments, rather than flattening out for low *T*, is caused by the downscaling of the lattice that is necessary in our theoretical model. In order to scale down the superconducting coherence length so that it remains comparable to the thickness of the superconducting layer, the on-site interaction must be increased, leading to a higher $T_C$. Since the temperature interval is larger, a smaller fraction of the temperatures exist in the low-temperature limit where the free energy is temperature independent. Keeping in mind that our measurements of $H_{\text {OOP}}$ show a dependence on the magnitude of the Rashba SOC, we can conclude that the SOC induced change in magnetic anisotropy below $T_C$ shown here must strongly contribute to the suppression in $H_{\text {OOP}}$ for the $10\times 10$  $\upmu\hbox {m}^2$ junctions.

## Conclusions

Our experiments point towards the superconductivity induced modification of the perpendicular magnetic anisotropy in the epitaxial Fe(001) films in the V(40 nm)/MgO(2 nm)/Fe(10 nm) system. The behaviour depends on the lateral dimensions of the junctions in the following way: First, for the smallest junctions, the magnetic field necessary for a full OOP magnetisation reorientation drops by an order of magnitude in the superconducting state, while for the rest of the junctions it varies only slightly. Second, in all but the largest junctions, an increase in the OOP misalignment angle between the soft Fe(10 nm) layer and the hard one is observed when the temperature is decreased below $T_C$ *without any applied field*. This spontaneous reorientation is similar for $10\times 10$ to $30\times 30$  $\upmu\hbox {m}^2$ junctions and disappears in the largest ones, suggesting that superconductivity could be affecting the competition between the IP and OOP anisotropies (which is more pronounced for the smaller junctions) rather than being the result of the reorientation taking place at the edges of the samples. The decreasing of $H_\text {OOP}$ transition field in the superconducting state, which could also be stimulated by the application of electric field changing the Rashba SOC, is consistent with the theoretical prediction^10^ of the absolute minimum of free energy corresponding to the OOP spin direction in SC/SOC/FM hybrids with competing (IP vs OOP) anisotropies just below $T_C$. The magnetostatic interaction between vortices and magnetic inhomogeneities could explain a weak hardening of the OOP transition in the largest junctions. A detailed theoretical analysis of the mutual interplay between the inhomogeneous magnetisation of the soft ferromagnet and the superconductor is, however, beyond the scope of this work. Our results open a route to active manipulation of perpendicular magnetic anisotropy in the expanding field of dissipation-free superconducting electronics involving spin^34–36^ or spin polarized supercurrents^37^.

## Methods

### Samples growth and characterization

The V(40 nm)/MgO(2 nm)/Fe(10 nm)/MgO(2 nm)/Fe(10 nm)/Co(20 nm) MTJ multilayer stacks have been grown by molecular beam epitaxy (MBE) in a chamber with a base pressure of $5\times 10^{-11}$ mbar following the procedure described in Ref.^38^. The samples were grown on (001) MgO substrates. A 10 nm thick seed of anti-diffusion MgO underlayer is grown on the substrate to trap the C from it before the deposition of the Fe (or V). The MgO insulating layer is then epitaxially grown by e-beam evaporation up to a thickness of approximately $\sim 2$ nm and the same process is then executed for the rest of the layers. Each layer is annealed at 450 °C for 20 min for flattening. After the MBE growth, all the MTJ multilayer stacks are patterned in micrometre-sized square junctions by UV lithography and Ar ion etching, controlled step-by-step in situ by Auger spectroscopy.

### Experimental measurement methods

The measurements are performed inside a $\hbox {JANIS}^{\tiny {\textregistered }}$ $\hbox {He}^3$ cryostat (the minimum attainable temperature is 0.3 K). The magnetic field is varied using a 3D vector magnet consisting of one solenoid (Z axis) with $H_\text {max}=3.5$ T and two Helmholtz coils (X and Y axis) with $H_\text {max}=1$ T. In our system the different magnetic states can be distinguished by looking at the resistance, so the relative orientation between two electrodes can be measured. The magnetoresistance measurements are performed by first setting the magnetic field to the desired value, then applying positive and negative current up to the desired voltage (5 mV unless otherwise stated), and averaging the absolute values of the measured voltage for the positive and negative current, obtaining a mean voltage which is used to calculate the resistance at that point. The temperature is measured and controlled with a LakeShore 340 thermometer.

## Supplementary Information

Supplementary Information.
