# Supplementary material for: Superconductivity assisted change of the perpendicular magnetic anisotropy in V/MgO/Fe junctions
Source: Sci Rep. 2021 Sep 24;11:19041. doi: 10.1038/s41598-021-98079-5 (PMC8463706; doi:10.1038/s41598-021-98079-5)
Supplement: Supplementary file 1 — Supplementary Information. [file 41598_2021_98079_MOESM1_ESM.pdf]

Supplementary Material: Superconductivity assisted change of the perpendicular magnetic anisotropy in V/MgO/Fe junctions

César González-Ruano[1], Diego Caso[1], Lina G. Johnsen[2], Coriolan Tiusan[3,4], Michel Hehn[4], Niladri Banerjee[5], Jacob Linder[2], and Farkhad G. Aliev[1,*]

[1]Departamento Física de la Materia Condensada C-III, Instituto Nicolás Cabrera (INC) and Condensed Matter Physics Institute (IFIMAC), Universidad Autónoma de Madrid, Madrid 28049, Spain
[2]Center for Quantum Spintronics, Department of Physics, Norwegian University of Science and Technology, NO-7491 Trondheim, Norway
[3]Department of Physics and Chemistry, Center of Superconductivity Spintronics and Surface Science C4S, Technical University of Cluj-Napoca, Cluj-Napoca, 400114, Romania
[4]Institut Jean Lamour, Nancy Universitè, 54506 Vandoeuvre-les-Nancy Cedex, France
[5]Department of Physics, Loughborough University, Epinal Way, Loughborough, LE11 3TU, United Kingdom
*farkhad.aliev@uam.es

ABSTRACT

The Supplementary material contains information about the calibration and correction of the superconducting coils alignment with the IP and OOP axis of the samples; the numerical simulations performed to evaluate the influence of Meissner effect, vortex-stray field magnetostatic interaction, microscopic defects and lateral size on the OOP reorientation behaviour; and details about the theoretical model supporting the experimental findings.

1 Correction of the OOP field misalignment

In order to ensure that the magnetic field is perfectly aligned with respect to the FM layers, we calibrate the angle between the V/MgO/Fe plane and the magnetic field created by the vector magnet superconducting coils by performing sub-gap conductance measurements at $T = 0.3$ K for different field directions around each axis. Figure S1 describes the calibration process in detail. The results are robust throughout the studied samples, with a misalignment of 8 ± 1 degrees with respect to the X-axis superconducting coil, which is accounted for in the OOP experiments. There is no observed in-plane misalignment (Y and Z axis coils).

For the highest OOP-AP transition fields measured (about 2000 Oe as shown in Figure 1c in the main text), the uncertainty of 1 degree in the misalignment calibration for the X coil could result in an IP component of the applied field of 35 Oe, which is about 20 times smaller than the IP coercive field of the hard FM layer[1]. Therefore, undesired IP field due to misalignment can be ruled out as an explanation for the observed AP transitions.

2 Numerical simulations

Micromagnetic simulations were carried out using the MuMax3[2] software in order to estimate (i) the possible influence of Meissner effect on the OOP transition dynamics, (ii) the role of magnetic inhomogeneities caused by defects on the volatility of the OOP magnetic state (characterized by the OOP to IP transition field, $H_{\text{OOP-IP}}$) and (iii) the OOP reorientation dynamics. Additional simulations of the OOP transition were performed varying the lateral size of the simulated samples, in order to contrast the results with the observed enhancement of the OOP transition field H_{OOP} with the junctions' lateral size. The following typical Fe magnetic parameters were implemented on the system: saturation magnetization $M_S = 2.13$ T (1.7×10^6 A/m), exchange stiffness $A_{\text{exch}} = 2.1 \times 10^{-11}$ J/m, damping $\alpha = 0.02$ and first order cubic anisotropy $K_{C1} = 4.8 \times 10^4$ J/m^3. All simulations were made with $T = 0$ K. The simulation cells of the system were set at 128 for the IP components and at 16 for the OOP component. The lateral size of the simulated samples was 0.4×0.4 μm^2, with a thickness of 10 nm. The system was additionally surrounded by a vacuum box of an added 100 nm (50 nm on each side) and 3 nm on the top and bottom of the

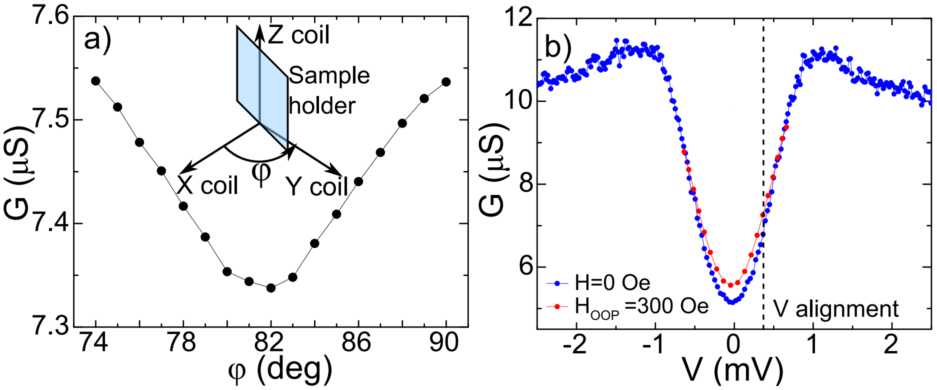

Figure 1. Calibration measurements for the X axis SC coil in a 20×20 μm^2 junction at $T = 0.3$ K. (a) shows the conductance measured at $V = 0.3$ mV, inside the SC gap, for different values of the φ angle (defined in the inset, which is a sketch of the sample holder situation with respect to the three SC coils). (b) shows two conductance curves at $H = 0$ and $H_{\mathrm{OOP}} = 300$ Oe. The dashed line indicates the applied voltage during the calibration process. When an OOP magnetic field is present, the SC gap diminishes and the conductance increases. The misalignment angle is therefore obtained as the one which minimizes the conductance ($\varphi = 82$ deg in (a)).

sample, leaving the discretization size of the simulation at $3.9 \times 3.9 \times 1$ nm. Higher discretization in the OOP direction has been chosen on purpose to observe the OOP effects with high accuracy in the simulations. Perpendicular magnetic anisotropy (PMA) was introduced on the top and bottom layers of the Fe as surface anisotropy, with a value of $K_{S1} = 8.32 \times 10^{-3}$ J/m^2. Due to computational limitations on the size and detail of the simulations, the results discussed in this section should be taken as qualitative support for the experimental results, rather than quantitative estimations.

2.1 Influence of Meissner effect on the OOP magnetization reorientation

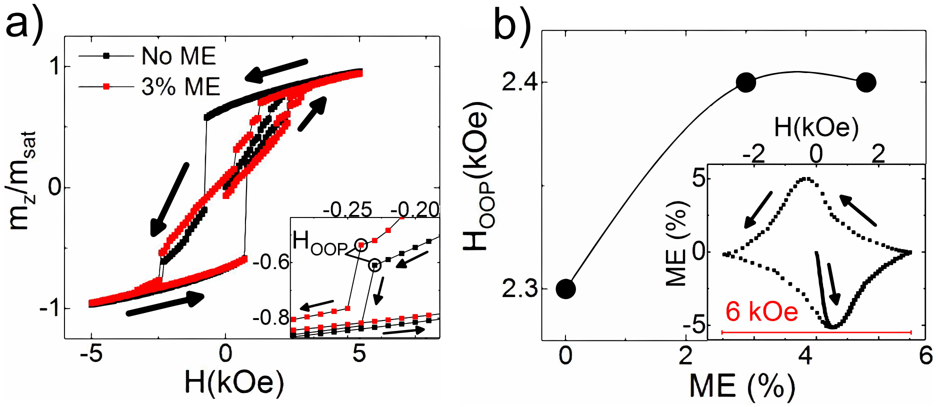

Figure 2. (a) Numerical simulations of the hysteresis loops reproducing the IP to OOP transition for different strengths of ME. (b) shows the simulated OOP transition field H_{OOP} as a function of the strength of the ME.

The influence of superconductivity on the OOP transition has been studied by performing micromagnetic simulations with MuMax3 for 10 nm thick, 0.4×0.4 μm^2 Fe(001) films under the influence of a superconducting vanadium layer. We simulated OOP hysteresis cycles where a correction to the applied field was added based on a typical Meissner effect (ME) hysteresis cycle (obtained from[3], shown in Figure S2b inset), scaled for different values of field contribution from Meissner effect and adapted to the first and second critical fields of vanadium (correspondingly H_{c1} and H_{c2}). The contribution from superconducting vortices was taken into account by using an in-group developed program that numerically solves the time dependent Ginzburg-Landau equations in order to simulate the behaviour of type II superconductors under magnetic fields[4]. The initial stray fields from an in-plane saturated FM simulation were used to generate a distribution of vortices, and then the fields generated by those vortices were calculated[5] and added into the corrected hysteresis cycle. Our simplified numerical

model, although limited, provides qualitative support for the mutual magnetostatic interaction between the FM and SC as the possible origin of the behaviour of the H_{OOP} field in the superconducting state. We also note that simulations with a contribution exceeding 7% of Meissner effect resulted in the OOP state being volatile in the hysteresis cycle (i.e. the magnetization returns to an IP configuration before returning to zero field), in contradiction with the experimental observations. Consequently, we have not considered larger contributions of Meissner effect as a possible explanation for the observed behaviour of H_{OOP}. We also underline that a complete numerical solution is a great challenge which is outside our current capabilities, as the problem should be solved self-consistently, so the results should be understood in a qualitative way.

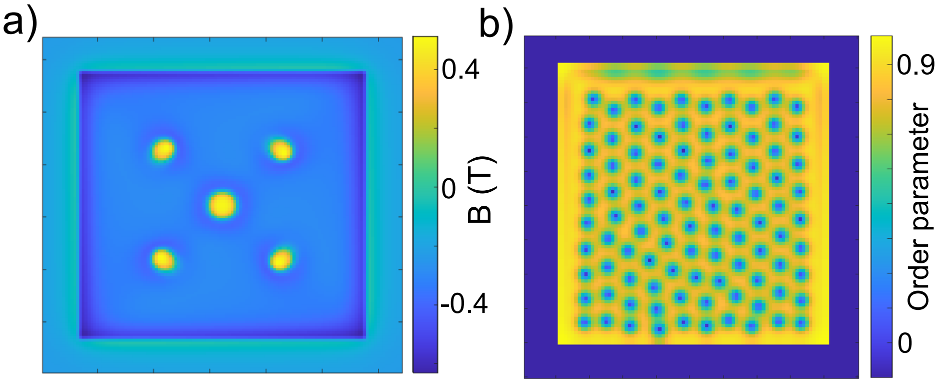

Figure 3. (a) Snapshot of the stray fields calculated in a micromagnetic simulation of a 0.4×0.4 micron Fe sample behaviour under OOP applied fields just before the OOP reorientation. Five domains can be observed with an OOP magnetization resulting in high stray fields, which trigger the reorientation in the rest of the film. (b) shows the stationary state reached in a superconducting simulation on a 0.4×0.4 micron vanadium layer at $T = 2$ K when stray fields are present at the edges of the sample. The vortices fill the interior of the film, which would result in higher OOP fields affecting the neighbouring FM layer and therefore triggering the OOP reorientation.

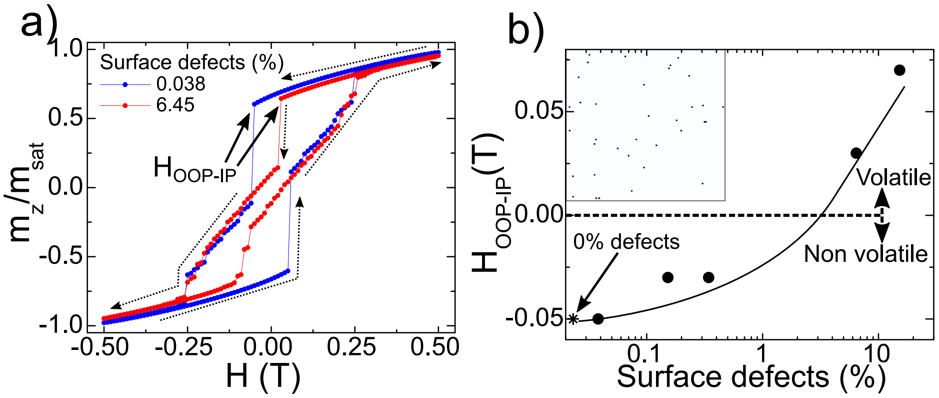

Figure 4. Influence of defects on the spin reorientation transition. (a) shows two OOP hysteresis cycles with different amounts of surface defects. The OOP-IP transition field is indicated by solid line black arrows, while the arrows with dotted lines mark the direction of the cycle. (b) plots the transition field $H_{OOP\text{-}IP}$ against the % of simulated defects (in log scale). An asterisk has been manually added with the transition field for the simulation with no defects. For simulations with more than 1% of defects, the OOP-IP transition becomes volatile (i.e. it happens before the field changes from positive to negative), in contradiction with the experimental results. The solid line is a guide for the eye, while the inset shows an example image of the surface defects introduced.

Another concern about the OOP reorientation is the possibility of it being a trivial effect produced at the edges of the FM layers. As mentioned in the main text, there is experimental evidence pointing against this possibility. However, a more thorough study has been performed in order to fully discard this scenario. First, we simulated the field-induced OOP reorientation in 0.4×0.4 micron Fe films. As shown in figure S3a, the reorientation seems to be triggered by OOP oriented domains in the

interior of the film.

On the other hand, we wanted to see the influence of any possible dominating edge effects on the underlying SC layer. Simulations of 0.4×0.4 micron V films were made using the code for type II superconductors described above at $T = 2$ K, with OOP magnetic fields applied at the edges of the simulated samples. As shown in figure S3b, the stationary state is reached when vortices fill the interior of the film. This would produce high OOP stray fields affecting the interior of the Fe layer, triggering a reorientation in the whole film rather than limiting it to the edges.

2.2 Influence of defects on the OOP-IP magnetization reorientation

In order to better understand the experimentally observed non-volatility of the OOP state in the junctions, we have simulated numerically the influence of randomly distributed surface magnetization defects within the bottom and top layers of the 10 nm thick Fe layer. The defects are introduced in the simulations as spots of enhanced surface saturation magnetization ($M_S(\text{defects}) = 1.25 \times M_S(\text{Fe})$). We have found that the introduction of a small number (about $10^{-3}\%$) of magnetic defects per layer does not affect the non-volatility, but only varies the characteristic field $H_{\text{OOP-IP}}$ of the transition from the OOP state to the IP alignment that takes place after the initial magnetization saturation (Figure S4). Above some critical defect number of about $2 \times 10^{-3}\%$ defects per layer the OOP-IP transition becomes volatile. As long as we always observe experimentally non-volatility of this transition in our junctions, we can conclude that there is a relatively small number of magnetic defects present in the epitaxial MTJs under study.

2.3 Dependence of H_{OOP} with the junctions lateral size

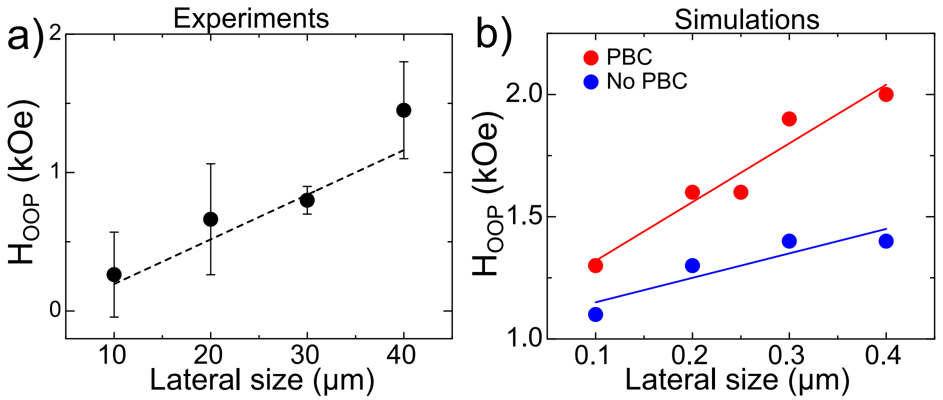

Figure 5. Dependence of the OOP transition field with the lateral size of the junctions. (a) shows experimental results, with the error bars corresponding to the standard deviation of the measured samples for each size. (b) shows the transition field for micromagnetic simulations of different lateral sizes. Note that, due to computational limitations, the simulated sizes are smaller than the actual samples. However, the trend is in qualitative agreement with the experimental results. The lines are linear fittings of the experimental/simulation points, which should serve as guides for the eyes. This trend is accomplished both with or without considering periodic boundary conditions (PBC).

The behaviour of the OOP transition field H_{OOP} has been studied as a function of the samples lateral size, in a total of 14 different samples varying from 10×10 to 40×40 μm^2. We found an increasing trend of H_{OOP} with the lateral size, as shown in Figure S5a. Micromagnetic simulations of the same transition have been performed in Fe films with lateral sizes of 0.1×0.1 to 0.4×0.4 μm^2 (as the real dimensions were computationally prohibitive to simulate) with a qualitative agreement to the experimental results, as shown in Figure S5b. These simulations have been made in the absence of previously discussed phenomena such as defects or Meissner effect.

3 Bogoliubov–de Gennes theoretical model

In order to demonstrate how the superconducting proximity effect can cause a change in the magnetic anisotropy, we consider a tight-binding Bogoliubov–de Gennes model for the V/MgO/Fe heterostructure. This model enables us to calculate the free energy of the system, so that we can study how the energy cost for reorienting the magnetization changes with temperature. We here only focus on the superconductivity-induced decrease in H_{OOP} observed for 10×10 μm junctions. Vortex formation and the size of the lattice is not taken into account here, and instead discussed in section S2.1 and in the microscopic model section

in the main text. For the microscopic model of the V/MgO/Fe heterostructure, we consider the Hamiltonian

$$H = -t\sum_{\langle i,j\rangle,\sigma} c^\dagger_{i,\sigma}c_{j,\sigma} - \sum_{i,\sigma}(\mu_i - V_i)c^\dagger_{i,\sigma}c_{i,\sigma} - \sum_i U_i n_{i,\uparrow}n_{i,\downarrow} + \sum_{i,\alpha,\beta} c^\dagger_{i,\alpha}(h_i\cdot\sigma)_{\alpha,\beta}c_{i,\beta} - \frac{i}{2}\sum_{\langle i,j\rangle,\alpha,\beta}\lambda_i c^\dagger_{i,\alpha} n\cdot(\sigma\times d_{i,j})_{\alpha,\beta}c_{j,\beta}. \quad (1)$$

The first term describes the nearest-neighbor hopping, where t is the hopping integral. The second term describes the chemical potential μ_i at each lattice site i, and the potential barrier $V_i > 0$ present in the insulating MgO layers. The third term gives rise to an attractive on-site interaction in the superconducting V layer described by the onsite potental $U_i > 0$. The fourth term introduces a local magnetic exchange field h_i giving rise to ferromagnetism in the Fe layer. The Pauli matrices are contained in the vector σ. The last term describes the Rashba spin-orbit coupling boosted by the MgO layers, where the spin-orbit field has a magnitude λ_i and is directed along the interface normal n. The vector $d_{i,j}$ connects site i and j. In the above Hamiltonian, $c^\dagger_{i,\sigma}$ and $c_{i,\sigma}$ are the second-quantization electron creation and annihilation operators at site i with spin σ, and $n_{i,\sigma} \equiv c^\dagger_{i,\sigma}c_{i,\sigma}$ is the number operator. The superconducting term is treated by a mean-field approach assuming that $c_{i,\uparrow}c_{i,\downarrow} \approx \langle c_{i,\uparrow}c_{i,\downarrow}\rangle + \delta$, where terms to the second order in the fluctuations δ are negligible. The superconducting gap is defined as $\Delta_i \equiv U_i\langle c_{i,\uparrow}c_{i,\downarrow}\rangle$ and must be treated self-consistently. The above model is valid in the ballistic limit, but since the effects considered here depend on the formation of s-wave odd-frequency triplets that are robust to impurity scattering we would obtain qualitatively the same results in the diffusive limit.

We consider a cubic lattice of size $N_x \times N_y \times N_z$ with an interface normal along the x axis. We assume periodic boundary conditions in the y and z directions, and apply the Fourier transform

$$c_{i,\sigma} = \frac{1}{\sqrt{N_y N_z}}\sum_k c_{i,k,\sigma}e^{\mathrm{i}(k\cdot i_{||})} \quad (2)$$

along these axes. To simplify notation we have defined $i \equiv i_x$, $j \equiv j_x$, $i_{||} = (i_y, i_z)$, and $k \equiv (k_y, k_z)$. We also use that

$$\frac{1}{N_y N_z}\sum_{i_{||}} e^{\mathrm{i}(k-k')\cdot i_{||}} = \delta_{k,k'}. \quad (3)$$

The Hamiltonian can be written on the form

$$H = H_0 + \frac{1}{2}\sum_k W^\dagger_k H_k W_k, \quad (4)$$

where the basis is given by

$$W^\dagger_k = [B^\dagger_{1,k},\dots,B^\dagger_{i,k},\dots,B^\dagger_{N_x,k}], \qquad B^\dagger_{i,k} = [c^\dagger_{i,k,\uparrow}\;\; c^\dagger_{i,k,\downarrow}\;\; c_{i,-k,\uparrow}\;\; c_{i,-k,\downarrow}], \quad (5)$$

and where the Hamiltonian matrix H_k consists of $N_x \times N_x$ blocks

$$H_{i,j,k} = \varepsilon_{i,j,k}\hat{\tau}_3\hat{\sigma}_0 + \delta_{i,j}\Big[\mathrm{i}\Delta_i\hat{\tau}^+\hat{\sigma}_y - \mathrm{i}\Delta^*_i\hat{\tau}^-\hat{\sigma}_y + h^x_i\hat{\tau}_3\hat{\sigma}_x + h^y_i\hat{\tau}_0\hat{\sigma}_y + h^z_i\hat{\tau}_3\hat{\sigma}_z - \lambda_i\sin(k_y)\hat{\tau}_0\hat{\sigma}_z + \lambda_i\sin(k_z)\hat{\tau}_3\hat{\sigma}_y\Big], \quad (6)$$

with row and column indices (i,j). Above, $\hat{\tau}_i\hat{\sigma}_j \equiv \hat{\tau}_i \otimes \hat{\sigma}_j$ is the Kronecker product of the Pauli matrices spanning Nambu and spin space, $\hat{\tau}^\pm \equiv (\hat{\tau}_1 \pm i\hat{\tau}_2)/2$, and

$$\varepsilon_{i,j,k} \equiv -2t\,[\cos(k_y)+\cos(k_z)]\,\delta_{i,j} - t(\delta_{i,j+1}+\delta_{i,j-1}) - (\mu_i - V_i)\delta_{i,j}. \quad (7)$$

The constant term is given by

$$H_0 = -\sum_{i,k}\left\{2t\,[\cos(k_y)+\cos(k_z)] + \mu_i - V_i\right\} + N_y N_z\sum_i \frac{|\Delta_i|^2}{U_i}. \quad (8)$$

By diagonalizing H_k, we obtain eigenvalues $E_{n,k}$ and eigenvectors

$$\Phi^\dagger_{n,k} = [\phi^\dagger_{1,n,k}\quad\cdots\quad\phi^\dagger_{N_x,n,k}], \qquad \phi^\dagger_{i,n,k} = [u^*_{i,n,k}\; v^*_{i,n,k}\; w^*_{i,n,k}\; x^*_{i,n,k}]. \quad (9)$$

The diagonalized Hamiltonian can be written as

$$H = H_0 - \frac{1}{2}\sum_{n,k}{}' E_{n,k} + \sum_{n,k}{}' E_{n,k}\gamma^\dagger_{n,k}\gamma_{n,k}, \quad (10)$$

where the marked sum goes over $\{n,k_y,k_z>0\}$, $\{n,k_y>0,k_z=0,-\pi\}$, and $\{n$ corresponding to $E_{n,k_y,k_z}>0, k_y=0,-\pi, k_z=0,-\pi\}$. Expectation values of the new operators can now be evaluated according to

$$\left\langle \gamma_{n,k}^{\dagger}\gamma_{m,k}\right\rangle = f(E_{n,k})\delta_{n,m}, \qquad \left\langle \gamma_{n,k}^{\dagger}\gamma_{m,k}^{\dagger}\right\rangle = \left\langle \gamma_{n,k}\gamma_{m,k}\right\rangle = 0, \tag{11}$$

where $f(E_{n,k})$ is the Fermi-Dirac distribution. The new quasi-particle operators are related to the old operators by

$$c_{i,k,\uparrow} = \sum_n u_{i,n,k}\gamma_{n,k}, \qquad c_{i,k,\downarrow} = \sum_n v_{i,n,k}\gamma_{n,k}, \qquad c_{i,-k,\uparrow}^{\dagger} = \sum_n w_{i,n,k}\gamma_{n,k}, \qquad c_{i,-k,\downarrow}^{\dagger} = \sum_n x_{i,n,k}\gamma_{n,k}. \tag{12}$$

The eigenenergies $E_{n,k}$ and eigenvectors $\Phi_{n,k}$ obtained in this diagonalization, can be used to calculate physical observables for the system. The superconducting gap is given by

$$\Delta_i = \frac{U_i}{N}\sum_{n,k}{}' \{u_{i,n,k}x_{i,n,k}^{*}[1-f(E_{n,k})] + v_{i,n,k}w_{i,n,k}^{*}f(E_{n,k})\}, \tag{13}$$

and is treated self-consistently.

We can calculate the critical field H_{OOP} for reorienting the magnetization from an IP to an OOP orientation. The Zeeman energy of an external magnetic field H is given by

$$F_{\mathrm{Zeeman}} = -\mu_0\mu_{\mathrm{tot}}\cdot H, \tag{14}$$

where μ_0 is the vacuum permeability, μ_{tot} is the total magnetic moment, and H is the applied field. If we consider a system where the free energy is minimal for an IP magnetization and maximal for an OOP magnetization, and we want to find the external magnetic field needed to reorient the magnetization to the OOP direction, we must require that $|F_{\mathrm{Zeeman}}| \geq F_{\mathrm{OOP}} - F_{\mathrm{IP}}$. We can then calculate the critical field from

$$H_{\mathrm{OOP}} = \frac{F_{\mathrm{OOP}} - F_{\mathrm{IP}}}{\mu_0\mu_{\mathrm{tot}}}. \tag{15}$$

To take into account other anisotropy contributions not covered by this model, we let $F_{\mathrm{OOP}} \to F_{\mathrm{OOP}} + K_{\mathrm{anis}}$. Above, the free energy is given by

$$F = H_0 - \frac{1}{2}\sum_{n,k}{}' E_{n,k} - \frac{1}{\beta}\sum_{n,k}{}' \ln(1+e^{-\beta E_{n,k}}), \tag{16}$$

where $\beta = (k_B T)^{-1}$. The total magnetic moment of the system for an OOP magnetization is given by

$$\mu_{\mathrm{tot}} = -2\mu_{\mathrm{B}}\sum_{i,n,k}{}' \left\{\mathrm{Re}(u_{i,n,k}^{*}v_{i,n,k})f(E_{n,k}) + \mathrm{Re}(x_{i,n,k}^{*}w_{i,n,k})\left[1-f(E_{n,k})\right]\right\}, \tag{17}$$

when the interface normal is directed along the x axis.

Since the lattice must be scaled down in order to make the system computationally manageable, we choose the magnitude of the on-site coupling potential U_i so that the superconducting coherence length is comparable to the thickness of the V layer. The superconducting coherence length is given by $\xi = \hbar v_F/\pi\Delta_0$, where $v_F = (1/\hbar)dE_k/dk\big|_{k=k_F}$ is the Fermi velocity calculated for the normal-state eigenenergy $E_k = -2t[\cos(k_x)+\cos(k_y)+\cos(k_z)] - \mu$, and Δ_0 is the zero-temperature superconducting gap.

We determine the superconducting critical temperature by a binomial search, where we decide if a given temperature is above or below T_C. This is decided by finding whether the superconducting gap measured in the middle of the superconducting region increases towards a superconducting solution or decreases towards a normal state solution from the initial guess $\Delta \ll \Delta(T=0)$ under iterative recalculations.

In Fig. 4 in the main text, we have used the parameters $t=1$, $\mu_{i\in\mathrm{S}} = 0.9$, $\mu_{i\in\mathrm{SOC}} = \mu_{i\in\mathrm{F}} = 0.8$, $V_{i\in\mathrm{SOC}} = 0.79$, $U = 1.4$, $\lambda = 0.4$, $h = 0.8$, $N_x^{\mathrm{S}} = 28$, $N_x^{\mathrm{SOC}} = 3$, $N_x^{\mathrm{F}} = 8$, and $N_y = N_z = 50$. This gives a coherence length of 21 lattice sites. All length scales are scaled by the lattice constant a, all energy scales are scaled by the hopping parameter t, and the magnitude of the spin-orbit coupling is scaled by ta.

References

1. C. González-Ruano, L. G. Johnsen, D. Caso, C. Tiusan, M. Hehn, N. Banerjee, J. Linder, and F. G. Aliev, Superconductivity-induced change in magnetic anisotropy in epitaxial ferromagnet-superconductor hybrids with spin-orbit interaction, Phys. Rev. B **102**, 020405(R) (2020).
2. A. Vansteenkiste, J. Leliaert, M. Dvornik, M. Helsen, F. Garcia-Sanchez and B. Van Waeyenberge, The design and verification of MuMax3, AIP Advances **4**, 107133 (2014).
3. R. Flükiger, Overview of Superconductivity and Challenges in Applications, Reviews of Accelerator Science and Technology **5**, 1-23, (2012).
4. A. Lara, C. González-Ruano and Farkhad G. Aliev, Time-Dependent Ginzburg-Landau Simulations of Superconducting Vortices in Three Dimensions, Low Temperature Physics/Fizika Nizkikh Temperatur, **46**, 4, 386-394 (2020).
5. A. M. Chang, H. D. Hallen, L. Harriott, H. F. Hess, H. L. Kao, J. Kwo, R. E. Miller, R. Wolfe and J. van der Ziel, Scanning Hall probe microscopy, Appl. Phys. Lett. **61**, 1974 (1992).
